# Necrotizing Urethritis due to *Aerococcus urinae*

**DOI:** 10.1155/2015/136147

**Published:** 2015-06-11

**Authors:** Abdulrahman A. Babaeer, Claudia Nader, Vito Iacoviello, Kevin Tomera

**Affiliations:** ^1^Urology Department, St. Elizabeth Medical Center, Brighton, MA 02135, USA; ^2^Department of Infectious Diseases, St. Elizabeth Medical Center, Brighton, MA 02135, USA

## Abstract

A 49-year-old male presented to the emergency with hematuria and pain in the shaft of the penis for one day. The patient was found to be in a state of shock. The shaft of the penis and the scrotum were swollen and tender. No skin necrosis was observed and no crepitus was palpable. Serum white count (WBC) was 29.5 × 10^3^/*μ*L. A CT scan showed gas in the corpus spongiosum. Antibiotics were started with IV metronidazole, vancomycin, and piperacillin/tazobactam. Metronidazole was then replaced by clindamycin. Exploration was performed but no necrotic tissue was identified. Cystourethroscopy revealed dusky looking urethra. A suprapubic tube and a urethral catheter were placed in the bladder. WBC trended down to 13.9 × 10^3^/*μ*L on the fourth postoperative day. Urine culture grew *Aerococcus urinae* and blood cultures grew *Alpha Hemolytic Streptococcus.* On the sixth day, the patient was feeling worse and WBC increased. MRI revealed absent blood flow to the corpus spongiosum. Urethroscopy revealed necrosis of the urethra. Urethrectomy was performed via perineal approach. The patient immediately improved. The patient was discharged on the sixth postoperative day to continue ampicillin/sulbactam IV every 6 hours for a total of 4 weeks from the day of urethrectomy.

## 1. Case Scenario

A 49-year-old African American male with bipolar disorder presented to the emergency department at another hospital in Boston, with gross hematuria and excruciating pain localized to the shaft of the penis that started one day prior to his presentation. In the previous two days, he was constipated and had to take a suppository laxative. The patient denied previous episodes of hematuria or pain. He had no history of urolithiasis. He denied any recent trauma to his penis. He had no urethral discharge and he denied any history of prior sexually transmitted disease. He is monogamous with his girlfriend and admitted having vaginal intercourse 6 days ago. His partner was recently hospitalized and was receiving intravenous (IV) antibiotics for* Methicillin Resistant Staphylococcus aureus* (MRSA) infection of the vertebral spine. The patient slept with her with the condom in place and woke up the next day with difficulty in urinating, which lasted for at least half of the day. It was not known that the patient had any medical condition and he was not receiving any form of treatment that could have affect his immune system.

At presentation, the patient was found to be in a state of shock with findings of multiple system organ failure. He was tachycardic with a heart rate (HR) of 135 beats per minute and hypotensive with a blood pressure (BP) of 77/48. Resuscitative measures were immediately initiated. The entire shaft of the penis and the scrotum were swollen and significantly tender. No skin necrosis was observed and no crepitus was palpable. Basic laboratory tests were done and serum white count (WBC) was 29.5 × 10^3^/*μ*L with 28% band neutrophil. Serum creatinine was 1.8 mg/dL; platelet count was as low as 58 × 10^3^/*μ*L with an INR of 1.8 and elevated liver enzymes, AST of 95 U/L, and ALT of 72 U/L. Blood was drawn and sent for culture. The patient partially responded to volume resuscitation. Since the patient did not have a classic picture of Fournier's gangrene, a CT scan of the pelvis was performed and showed gas in the corpus spongiosum at the level of the root of the penis. A polymicrobial necrotizing infection was suspected. Antibiotics coverage was started with IV metronidazole, vancomycin, and piperacillin/tazobactam.

The patient was then transferred to our hospital for further management. Metronidazole was replaced by clindamycin, and piperacillin/tazobactam and vancomycin were continued. Informed consent was obtained and a suprapubic tube (SPT) was placed. Urine was obtained at the time of SPT placement and sent for culture. This was followed by penoscrotal exploration performed at the level of the gas that was seen in the CT scan. However, no necrotic tissue was identified. Cystourethroscopy was performed afterwards and revealed dusky looking urethra with slough tissue. A urethral catheter was placed in the bladder over a guidewire.

The patient spent the night intubated in the ICU. He required inotropic support for few hours. After 10 hours from the surgery, he was hemodynamically stable and got extubated. The patient was managed afterwards on the regular floor and continued to be hemodynamically stable. His WBC trended down and reached a nadir of 13.9 × 10^3^/*μ*L on the fourth postoperative day. Urine culture was initially reported to grow* Alpha Hemolytic Streptococcus*; the organism was later identified as* Aerococcus urinae*. His blood cultures grew* Alpha Hemolytic Streptococcus* in 4 out of 4 bottles. A transthoracic echocardiogram was performed and did not reveal any vegetation.

**Figure 1:**
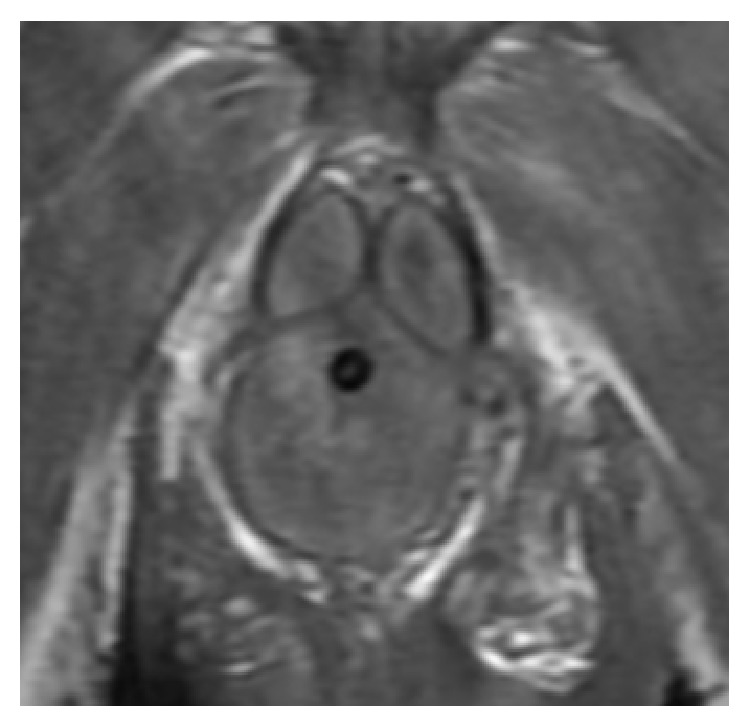
A representative MRI T1 image without gadolinium showing a coronal cut of the base of the penis with both corpora cavernosa and the corpus spongiosum having similar signal intensity.

**Figure 2:**
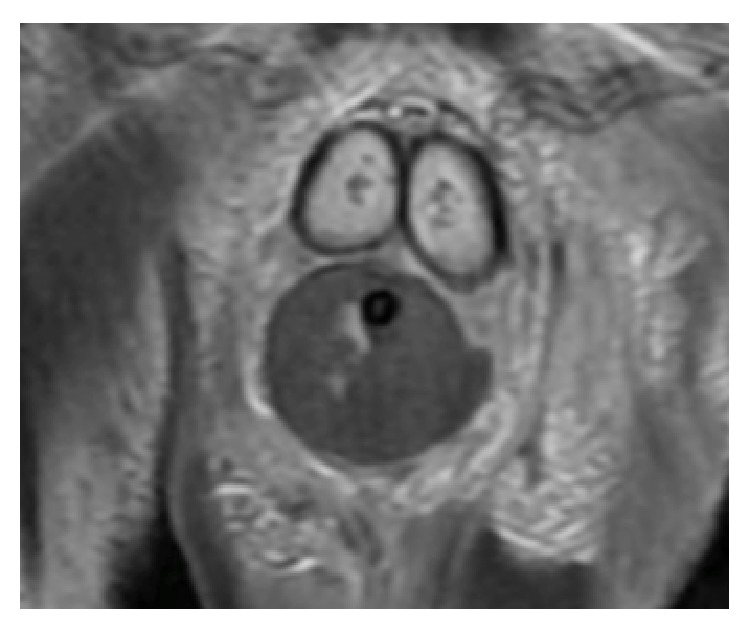
A representative MRI T1 image after gadolinium showing a coronal cut of the base of the penis with both corpora cavernosa having high signal intensity due to gadolinium uptake whereas the corpus spongiosum does not uptake gadolinium.

Despite broad-spectrum antibiotics, the patient was still feeling unwell. On the sixth day after surgery, WBC started to increase again and reached 27.7 × 10^3^/*μ*L; at that time, the patient reported feeling worse. His genitalia continued to be swollen and tender. An MRI of the penis was performed and revealed diffuse enlargement of corpus spongiosum along its extent and absent blood flow.  Figure 1 shows both corpora cavernosa and corpus spongiosum on T1 before injecting gadolinium.  Figure 2 shows enhancement of the corpora cavernosa with gadolinium but no enhancement of the majority of the corpus spongiosum and urethra. This finding was suggestive of absent blood flow to the corpus spongiosum and urethra. Subsequently, an informed consent for urethrectomy was obtained from the patient. Urethroscopy was performed and revealed necrosis of the urethra. Urethrectomy was performed via perineal approach using an inverted “Y” incision. The urethra, along with the periurethral soft tissue and corpus spongiosum, was excised from the pelvic membrane to the fossa navicularis. The wound was reapproximated in simple interrupted fashion and a half-inch Penrose drain was left for drainage.

The patient immediately improved afterwards. The tissue specimen grew also* Alpha Hemolytic Streptococcus*. Clindamycin was continued, piperacillin/tazobactam was switched to ampicillin/sulbactam, and vancomycin was discontinued. The Penrose drain was removed on the third postoperative day. The WBC fell and reached normal levels at 10.8 × 10^3^/*μ*L by the fifth postoperative day. Peripherally Inserted Central Catheter (PICC) was placed to continue antibiotics in the form of ampicillin/sulbactam IV every 6 hours for a total of 4 weeks from the day of urethrectomy. The patient was discharged home on the sixth postoperative day in a stable condition.

## 2. Discussion

To our knowledge, there has not been a reported case with necrotizing urethritis due to* Aerococcus urinae.* Necrotizing soft tissue infections (NSTIs) are fulminant infections affecting the soft tissue compartment and leading to widespread necrosis, systemic toxicity, and a high mortality rate if not treated early. Treatment principles are fluid resuscitation and correction of electrolyte and acid-base imbalance, early initiation of antibiotics, surgical debridement of the affected area, and supportive measures for organ failure [1, 2].

Fowler et al. in 1979 described a case where a 56-year-old female was diagnosed to have necrotizing urethritis secondary to Wegener's granulomatosis. The patient was successfully treated by excising the necrotic urethral and periurethral soft tissue followed by prednisone and cyclophosphamide [3].

*Aerococci *are gram-positive cocci that had been considered to be uncommon pathogens in humans. However,* Aerococcus urinae *and* Aerococcus sanguinicola *are now known to cause urinary tract infections and invasive infections.

*Aerococcus urinae* was first recognized as a separate species in 1992 [4]. Its normal habitat remains unclear. Interestingly, it shares characteristics with* Streptococci*,* Staphylococci, *and* Enterococci*.* Aerococcus urinae* exhibits alpha hemolysis on blood agar, grows in clusters, and is intrinsically resistant to sulfonamide [5]. Unlike* Staphylococcus*, it does not produce catalase. Its growth occurs under both aerobic and anaerobic conditions. Not surprisingly, it can be commonly misidentified as an alpha hemolytic* Streptococcus* on microbiology reports. Proper identification of* Aerococcus urinae *can be done with biochemical methods; however sequencing of the gene coding 16S rRNA remains the gold standard for species determination [6].

In animal models of streptococcal infection, clindamycin has been shown to yield response rates superior to those of penicillin, even in the context of delayed treatment [7].

*Aerococcus urinae* has very low MICs for penicillin and somewhat low MICs cephalosporins, whereas their MICs to aminoglycosides are high. It is generally sensitive to vancomycin; however elevated MICs have been reported [8]. The organism was thought to be generally sensitive to ciprofloxacin and levofloxacin; however acquired resistance has been described lately, which makes the choice of empiric quinolones for treating* Aerococcus urinae* doubtful [9, 10].

*Aerococcus urinae *can colonize the urinary tract. Not only has it been implicated in urinary tract infections, but also it has been reported as a cause of invasive infections such as bloodstream infections, infectious endocarditis, and spondylodiscitis [11–13]. Patients with severe* Aerococcus* infections were mostly elderly males who had some underlying urologic abnormalities such as benign prostatic hypertrophy [14]. Among those who developed infectious endocarditis, the mortality rate was high. Biofilm formation and platelet aggregation are believed to contribute to the bacterial virulence of this organism [15].

We believe that the patient might have acquired this infection through a sexual encounter with his partner who was being treated with IV antibiotics that could have changed her vaginal flora. Also, the fact that the patient slept with the condom in place could have provided an excellent medium for the organism to multiply and thus result in a fulminating infection. However, this cannot be confirmed based on the available information.

We report to the best of our knowledge the first case of severe* Aerococcus urinae* urinary tract infection with necrotizing urethritis in a relatively young man. The severity of his clinical presentation and the extent of the urethral infection emphasize what has been reported in the literature about the virulence of this organism. Furthermore, the patient showed no clinical improvement on antibiotics alone and unfortunately required a urethrectomy.
